# Impact of *Lactiplantibacillus plantarum* on the fermentation quality, nutritional enhancement, and microbial dynamics of whole plant soybean silage

**DOI:** 10.3389/fmicb.2025.1565951

**Published:** 2025-05-19

**Authors:** He Meng, Yuwen Xu, Lin Wang, Jianping Wang, Bing Wang, Huimin Wu, Donghui Hou, Sui Wang, Xiaohong Tong, Yan Jiang, Shaodong Wang

**Affiliations:** ^1^Agricultural College, Northeast Agricultural University, Harbin, China; ^2^Key Laboratory of Soybean Biology of Chinese Education Ministry, Northeast Agricultural University, Harbin, China; ^3^Beidahuang Group Heilongjiang Tangyuan Farm Co., Ltd., Jiamusi, China; ^4^Heilongjiang Academy of Agricultural Sciences, Institute of Agricultural Product Quality and Safety, Harbin, China; ^5^National Key Laboratory of Smart Farm Technologies and Systems, Harbin, China; ^6^Heilongjiang Academy of Green Food Science/National Soybean Engineering Technology Research Center, Harbin, China

**Keywords:** soybean, silage, *Lactiplantibacillus plantarum*, bacterial community, metabolites

## Abstract

Soybean (*Glycine max* (L.) Merr.) is an important leguminous crop with rich nutrients and wide uses, yet soybean straw is often treated as waste in many areas without sufficient regard for its nutritional value. For the sustainable utilization of biomass resources, this study assessed the fermentation quality, microbial communities, and metabolites of whole plant soybean (WPS) silage with and without *Lactiplantibacillus plantarum* (LP) over different fermentation periods of 7, 15, 30, 60, and 90 days. With LP, there was a significant increase in dry matter (DM), crude protein (CP), and water-soluble carbohydrate (WSC) content of silage (*p* < 0.01) and a significant decrease in pH (*p* < 0.01). Incorporating LP into WPS silage significantly elevated lactic acid (LA) concentration, thereby improving fermentation quality. 16S rRNA gene sequencing revealed that LP inoculation significantly altered bacterial diversity and composition, notably increasing the relative abundance of *Lactobacillus* and promoting beneficial shifts in the microbial community during silage fermentation. Notably, LP treatment significantly promoted lysine biosynthesis, a key essential amino acid pathway, thereby contributing to the nutritional enhancement of the silage. Results showed that adding LP to WPS at ensiling can improve silage microbial community structure optimize metabolic processes, produce superior metabolites, and enhance the silage’s fermentation quality and nutritional value, after 60 days of fermentation. In summary, WPS silage with LP addition could serve as a promising strategy for preserving high-protein forage silage.

## Graphical abstract

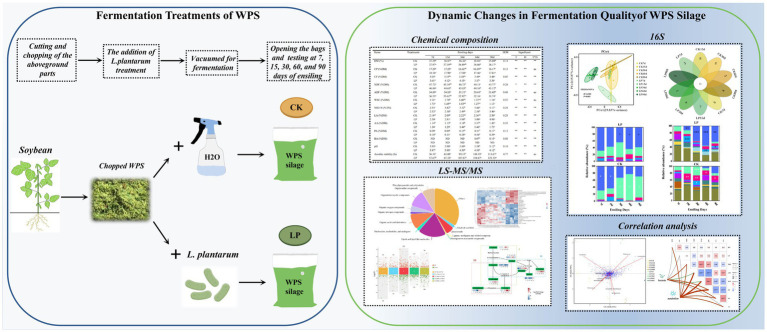

## Introduction

With the rapid development of the economy in the developing country, there has been a substantial surge in the demand for animal-derived products, consequently exacerbating the inadequacy in the supply of environmentally sustainable protein feed. Additionally, the limited arable land in some developing countries hinders widespread cultivation of some high protein forage grasses (Lin et al., 2017). To solve this issue, it is imperative to diversify protein forage sources and advance the development of high protein forage materials. Soybean (*Glycine max* (L.) Merr.) is a rich protein legume species with economic significance (Jahanzad et al., 2016). Forage soybeans are widely grown in nations such as Turkey and the United States, recognized for their high forage value (Chang et al., 2012) and manageable resource at its maturity stage (Wang et al., 2021b). The soybean straw with rich lignocellulosic is currently underutilized and significantly wasted as a major agricultural residue. Therefore, the whole soybean plant (WPS) silage can not only solve the shortage of high protein feed, but also promote the effective utilization of soybean straw resources. Moreover, even when cultivated in the arid and saline-alkaline soils, the crude protein (CP) content of WPS silage is sufficient to meet the daily nutritional requirements of ruminant animals (Wang W. N. et al., 2022). WPS with low seed yield in some barren soil and saline-alkali soil is effectively utilized as a new biomass resource, which can alleviate land use pressure and mitigate the shortage of high-protein feeds. Also, soybean as an emergency crop was grown in the damaged soil under natural calamities, providing livestock with incompletely mature WPS silage during the suitable cutting harvest periods, thus maximizing reduction of economic losses. Therefore, WPS is a promising and cost-effective forage resource for cleaner production of high protein silage feed without residues.

Ensiling, a microbial-driven anaerobic fermentation process, is a well-established method for preserving moist forage. It relies on lactic acid bacteria (LAB) to produce organic acids, lower pH levels, and inhibit the growth of yeasts, fungi, and competing bacteria, ensuring a year-round availability of palatable forage for ruminants (Weinberg and Muck, 1996). The advantage of soybean silage as animal feed lies in its rich protein content, reducing reliance on market fluctuations in protein prices. However, the natural ensiling of soybean is hindered by its high buffering capacity, limited water soluble carbohydrate (WSC) content, and insufficient epiphytic LAB count, resulting in silage fermentation failure and high butyric acid (BA) content (Yang et al., 2016). Therefore, it is essential to continue developing reliable methods to enhance the fermentation quality of soybean silage. Silage additives can effectively enhance the fermentation quality of forage, with LAB being extensively utilized in recent years, particularly in alfalfa silage where similar improvements have been observed (Huo et al., 2021). LAB, which includes genera such as *Lacticaseibacillus*, *Lactiplantibacillus*, *Levilactobacillus*, *Lactococcus*, *Leuconostoc*, *Pediococcus*, and *Streptococcus* (Cichońska et al., 2022) are commonly used as additives to improve the quality of silage. As an additive intended to improve the quality of silage, *Lactiplantibacillus plantarum* was widely used (Si et al., 2023). A study demonstrated that using *Lactiplantibacillus plantarum* as an additive in Italian ryegrass silage improved its fermentation quality, leading to higher lactic acid (LA) and lower BA production (Yan et al., 2019). Ni et al. (2017) reported that the combined addition of *Lactiplantibacillus plantarum* and *Pediococcus pentosaceus* as additives significantly improved the fermentation quality of soybean silage by enhancing the presence of beneficial *Lactobacillus* and reducing undesirable microorganisms.

To the best of our knowledge, no studies have specifically focused on the impact of *Lactiplantibacillus plantarum* alone as a silage additive on the fermentation characteristics and microbial community of WPS silage. Silage quality is largely determined by the composition and succession of the microbial community, and which further impacts fermentation metabolites (Zhao et al., 2021). We hypothesized that the inoculation with *Lactiplantibacillus plantarum* would improve fermentation characteristics, promote beneficial microbial succession, and reshape metabolite profiles of WPS silage. This study focused on metabolites and microbial diversity of WPS silage, comparing samples with and without *Lactiplantibacillus plantarum* by utilizing the Illumina NovaSeq 6000 for bacterial analysis and LC–MS/MS for metabolite detection, to improve the high quality and efficiency utilization of WPS forage as a new forage resource and clean production.

## Materials and methods

### Silage preparation

The soybean variety “Dongnong Si Dou 1” was sown on May 20, 2022, at the experimental field of the Northeast Agricultural University, Harbin, China (126^°^3′30″E, 45^°^44′34″N, elevation 178 m), with a density of 250,000 plants/ha and a basal application of compound fertilizer (N: P_2_O_5_: K_2_O = 45:75:75) at 225 kg/ha. The whole plant soybean during the early grain filling stage was manually harvested on August 17, 2022, leaving a 15 cm stubble. Following harvest, the plants was wilted outdoors for 8 h on a clean sheet, then chopped to 2–3 cm lengths by a forage chopper (YL100L-2, Weihai, China), mixed thoroughly, and treated with *Lactiplantibacillus plantarum* CGMCC1.557 (LP, 1 × 10^6^ cfu/g fresh matter, Haibo Biotechnology Co., Ltd., Qingdao), similar to the method described by Xu et al. (2020). In contrast, the control group received a treatment of sterile water. Each portion of the material was weighed 500 g and was packed in polyethylene bags (25 cm × 30 cm, Wenzhou, China) and sealed with a vacuum sealer (DZQ-420 C, Anshengke, Quanzhou, China). A total of 30 bags (5 ensiling stages × 2 treatments × 3 replicates) were stored at 20–25°C in the darkness. Samples were collected from the 30 bags at 7, 15, 30, 60, and 90 days for the analysis of fermentation characteristics, bacterial community, and metabolites. The samples were organized into two main groups based on the treatment: control (CK) and *Lactiplantibacillus plantarum* (LP).

### Chemical composition and fermentation characteristics analysis

Upon reaching the designated fermentation durations, bags were opened for sampling. Each silage ensiling sample was weighed 100 g and dried at 65°C for 48 h to ascertain the DM content, then ground through a 1 mm sieve and stored at room temperature in a desiccator before chemical analysis. The water soluble carbohydrates (WSC) content were measured according to the thracenone–sulfuric acid method, ether extract (EE) and CP contents were measured using the Soxhlet extraction method and the Kjeldahl method, respectively (AOAC, 1990). The acid detergent fiber (ADF) and neutral detergent fiber (NDF) was analyzed using the method of Van Soest et al. (1991) determined according to the methods by Broderick and Kang (1980). A sample of 10 g was taken from each silage fermentation bag, mixed with 100 g of distilled, sterilized water, and homogenized for 35 s. The mixture was subsequently filtered through medical gauze, and the We measured pH immediately afterward. The homogenate was centrifuged at 8,000 × g for 10 min at 4°C. The aqueous extract was analyzed for organic acids using high-performance liquid chromatography (HPLC, Primaide, Hitachi, Tokyo, Japan) with a 0.22 m membrane, a UV detector at 210 nm wavelength, a column temperature of 50°C, and a mobile phase of methanol and 0.01 mol/L potassium dihydrogen phosphate (pH 3.5, 0.7 mL/min, 50°C). The aerobic stability (AS) of the silage was assessed by recording the number of hours required for the silage temperature to rise 2°C above the ambient temperature, with the test conducted at a room temperature of 25°C (Wilkinson and Davies, 2013).

### Microbial diversity analysis

According to the manufacturer’s guidelines, total bacterial genomic DNA was extracted using Fast DNASPIN kits (MP Biomedicals, Santa Ana, CA, United States) and preserved at −20°C before additional analyses. PCR amplification of the V3-V4 region of bacterial 16S rRNA genes was conducted using the primers 338F (5′-ACTCCTACGGGAGGCAGCA) and 806R (5′-GGACTACHVGGGTWTCTAAT). Standard sequencing protocols were employed on the Illumina NovaSeq platform with pair-end 2 × 300 bp sequencing by Biomarker Technologies Co., Ltd. The raw reads were deposited in the National Center for Biotechnology Information (NCBI) database under accession number PRJNA1085090. In this study, the DADA2 (Callahan et al., 2016) method in QIIME2 2020.6 was employed for denoising paired-end sequences, merging them, and removing chimeric sequences to produce final non-chimeric reads, which were then assigned into amplicon sequence variants (ASVs). Taxonomic classification at the phylum and genus levels was performed based on the SILVA database.^1^

### Metabolite analysis

Silage samples were ground post-freeze-drying and extracted using 70% aqueous methanol at 4°C overnight. The extracts were then centrifuged (12,000 rpm, 10 min) and filtered through a 0.22 μm membrane before LC–MS/MS analysis (Wang et al., 2021a). Metabolite analysis was performed using the MetWare database by Wekemo Tech Group Co., Ltd., Shenzhen, with VIP ≥ 1.0 and FC ≥ 2.0 as the criteria for differential metabolite selection. Metabolites were annotated using the Kyoto Encyclopedia of Genes and Genomes (KEGG) compound database and mapped to the KEGG pathway database.^2^

### Statistical analysis

Statistical analyses were performed using SPSS 26.0. Two-way ANOVA was conducted treatments effect with *Lactiplantibacillus plantarum* (T), ensiling days effect (D), and their interaction (T × D) as fixed factors. When significant interactions were found (*p* < 0.05), Tukey’s HSD test was used for *post-hoc* comparisons. Data are presented as mean ± SEM, with significance set at *p* < 0.05. Sequence data’s bioinformatics and statistical analyses were primarily conducted with QIIME2 2023.5 and R (V4.2.3). The significant variations in the abundance at the phylum and genus levels were discerned by two-sided Student’s *t*-test. Principal Coordinates Analysis (PCoA) and Variance partitioning analysis (VPA) were performed with the “Vegan” R package. In metabolomics, the “CMS” package facilitated peak identification, filtration, and alignment. Linear Discriminant Analysis Effect Size (LEfSe), Partial Least Squares Discriminant Analysis (PLS-DA), Canonical Correspondence Analysis (CCA), Short Time-series Expression Miner (STEM) analysis, Mantel test, as well as the creation of Petals diagrams, Circos maps, stacked bar charts, chord diagrams, and Volcano plots were conducted using OmicShare tools, a complimentary online platform for data analysis.^3^

## Results

### Chemical composition of WPS

As shown in Table 1, the chopped WPS sample exhibits a DM content of 36.26% and a CP content of 18.18%. The DM content is about 4.59% higher than the value reported by Zeng et al. (2020), while the CP content is approximately 3% higher. In comparison, the ADF content is 1.93% higher, and the NDF content is 0.3% lower than the values reported by Zeng et al. (2020).

**Table 1:** Chemical composition of the fresh WPS before ensiling.

Items	WPS	SEM
DM (%)	36.26	0.16
CP (%DM)	18.18	0.17
ADF (%DM)	33.32	0.52
NDF (%DM)	47.65	0.44
EE (%DM)	3.77	0.05
WSC (%DM)	3.84	0.55

WPS, whole plant soybean; DM, dry matter; CP, crude protein; ADF, acid detergent fiber; NDF, neutral detergent fiber; EE, ether extract; ND, not detected; WSC, water-soluble carbohydrate; SEM, standard error of means.

### Chemical composition and fermentation characteristics of WPS silage

In Table 2, a significant two-way interaction between *Lactiplantibacillus plantarum* (T) and ensiling duration (D) was observed for all indices (*p* < 0.01), except for the concentrations of CP, WSC, and ADF. In CK and LP groups, the contents of DM, WSC, CP, and pH decreased significantly (*p* < 0.01) as ensiling days increased. Both the LP group and the CK group exhibited the highest DM contents at 7 days of ensiling, 37.97 and 37.29%, respectively. The CK group showed the highest WSC content of 2.11 at 7 days of ensiling, whereas the lowest WSC content in the LP group was 1.13 at 90 days of ensiling. At 7 days of ensiling, the CP content reached its peak at 17.29% in the CK group and 18.16% in the LP group. As ensiling progressed, there was a statistically significant reduction (*p* < 0.01) in the levels of ADF and NDF in both the CK and LP groups. The LP group also exhibited a significant decrease in ADF and NDF content compared to the CK group (*p* < 0.05). The highest ADF and NDF contents in both groups were recorded at 7 days of ensiling. In the CK group, the NDF content was lower at 60 days (44.11%), while the lowest ADF content occurred at 60 days (30.41%). In the LP group, the lowest ADF content was recorded at 60 days (32.16%) and at 90 days (31.47%). There was no notable change in NDF contents across LP15d, LP30d, LP60d, and LP90d, all of which were lower than those at 7 days.

**Table 2:** Chemical composition, organic acid contents, pH, and aerobic stability of ensiled WPS.

Items	Treatments	Ensiling days					SEM	Significant		
		7d	15d	30d	60d	90d		T	D	T*D
DM (%)	CK	37.29^a^	36.87^a^	36.36^a^	36.01^a^	35.00^b^	0.14	**	**	**
	LP	37.97^a^	37.19^ab^	36.89^ab^	36.86^b^	36.17^c^				
CP (%DM)	CK	17.29^a^	17.25^a^	16.62^ab^	16.69^ab^	16.17^c^	0.13	**	**	ns
	LP	18.16^a^	17.96^a^	17.88^a^	17.46^a^	17.01^a^				
EE (%DM)	CK	3.05^c^	3.15^bc^	3.18^bc^	3.49^a^	3.48^a^	0.07	**	**	**
	LP	3.61^b^	4.12^a^	4.15^a^	3.17^c^	3.38^c^				
NDF (%DM)	CK	47.72^a^	45.34^ab^	46.75^b^	44.11^c^	43.77^c^	0.28	*	**	**
	LP	46.84^a^	44.65^b^	43.83^b^	44.16^b^	45.12^b^				
ADF (%DM)	CK	34.89^a^	34.83^a^	31.31^b^	30.41^b^	31.45^b^	0.41	*	**	ns
	LP	36.53^a^	35.62^ab^	32.92^bc^	32.16^c^	31.74^c^				
WSC (%DM)	CK	2.11^a^	1.78^b^	1.60^bc^	1.37^cd^	1.16^d^	0.57	**	**	ns
	LP	1.73^a^	1.45^ab^	1.53^ab^	1.27^bc^	1.13^c^				
NH_3_-N (%TN)	CK	2.53^c^	3.82^b^	3.52^b^	5.60^a^	5.17^a^	0.21	**	**	**
	LP	2.52^b^	2.58^b^	2.49^b^	2.38^b^	3.46^a^				
LA (%DM)	CK	2.18^ab^	2.09^b^	2.22^ab^	2.36^ab^	2.50^a^	0.21	**	**	**
	LP	2.50^c^	2.81^c^	3.98^b^	5.09^a^	4.98^a^				
AA (%DM)	CK	1.14^b^	1.13^b^	1.18^b^	1.17^b^	1.43^a^	0.35	**	**	**
	LP	1.34^c^	1.29^c^	1.44^b^	1.46^b^	1.73^a^				
PA (%DM)	CK	0.09^c^	0.09^c^	0.15^b^	0.11^b^	0.17^a^	0.13	**	**	**
	LP	0.18^b^	0.11^c^	0.28^a^	0.16^b^	0.29^a^				
BA (%DM)	CK	ND	ND	ND	0.07^b^	0.15^a^	0.01	**	**	**
	LP	ND	ND	ND	ND	ND				
pH	CK	5.95^a^	5.98^a^	5.89^a^	5.38^b^	5.12^b^	0.10	**	**	**
	LP	5.87^a^	5.88^a^	4.30^b^	4.10^b^	4.12^b^				
Aerobic stability (h)	CK	56.33^c^	61.00^c^	99.33^b^	108.33^a^	111.67^a^	4.77	**	**	ns
	LP	57.67^d^	67.33^c^	107.67^b^	118.67^a^	121.33^a^				

DM, dry matter; CP, crude protein; ADF, acid detergent fiber; NDF, neutral detergent fiber; EE, ether extract; WSC, water-soluble carbohydrate; NH_3_-N, ammonium nitrogen; LA, lactic acid; AA, acetic acid; PA, Propionic acid; BA, butyric acid; ND, not detected; SEM, standard error of means; CK, control with no inoculations; LP, silages inoculated with *Lactiplantibacillus plantarum*; 7d, 7 days of ensiling; 15d, 15 days of ensiling; 30d, 30 days of ensiling; 60d, 60 days of ensiling; 90d, 90 days of ensiling; ns, not significant. Means with different superscript letters in the same row (a–d) indicate significant differences (*P* < 0.05) based on Tukey’s HSD test. Asterisks in the “Significant” column represent statistical significance from the two-way ANOVA: *P < 0.05, **P < 0.01, and ns (not significant), corresponding to treatment (T), ensiling day (D), and their interaction (T × D).

A significant reduction (*p* < 0.01) in the levels of ADF and NDF both the CK and LP groups (Table 2). The LP group also showed a significant decrease in ADF and NDF content compared to the CK group (*p* < 0.05). The highest ADF and NDF contents in the CK and LP groups were at the 7 days of ensiling. In the CK group, the NDF contents was lower at 60 days (44.11%), while the lowest ADF contents occurred at 60 days (30.41%) of ensiling. In the LP group, the lowest ADF content was at 60 days (32.16%) and 90 days (31.47%). There was no notable change in NDF contents at LP15d, LP30d, LP60d, and LP90d, all of which were lower than that at 7 days. Previous studies have shown that low levels of ADF and NDF concentrations are associated with higher quality feeds and higher dry matter intake (Ni et al., 2014). Notably, the CK and LP groups showed the lower contents of ADF and NDF at 60 days, indicating they have a higher feed value compared to other fermentation periods. The decrease in crude fiber content could be attributed to the enzymatic activity of microorganisms, which break down the fiber during the fermentation process (Chen et al., 2022). The EE content in the CK group showed a significant increasing trend with the increase of silage days (*p* < 0.01), with the highest content (3.49%) at 60d of silage fermentation content was more than that in the CK group. This may be due to the microbial community change and reduction of lipolytic bacteria with the addition of LP.

The content of NH_3_-N increased significantly (*p* < 0.01) with the increase of ensiling days. The NH_3_-N content in the silage showed a significant (*p* < 0.01) reduction when treated with *Lactiplantibacillus plantarum*. A high content of NH_3_-N in the silage indicates significant protein degradation, which is attributed to slow acidification or clostridial activity (Ogunade et al., 2017). In this study, the addition of LP improved the fermentation process in the silage, as the NH_3_-N content of all silage treatments (<5% TN) was consistent with good-quality silage (<10% TN) (Mu et al., 2020). A characteristic of high-quality silage feed is its high concentration of LA (Maneerat et al., 2015). LA and AA are the primary organic acids contributing to the pH reduction in silage. In both the CK and LP groups, the LA content significantly increased (*p* < 0.01) as the ensiling period was extended. The LP group had the highest LA concentration, reaching 5.09% at 60 days of ensiling, indicating an improvement in the fermentation quality during the later stages of ensiling. At 90 days of ensiling, the AA concentrations significantly increased in both the CK and LP groups, indicating that the later stage of the silage process was beneficial for heterofermentation pathways, leading to an increase in AA levels.

### Bacterial community of WPS silage

#### Alpha-diversities and beta-diversity of WPS silage

**Figure 1:**
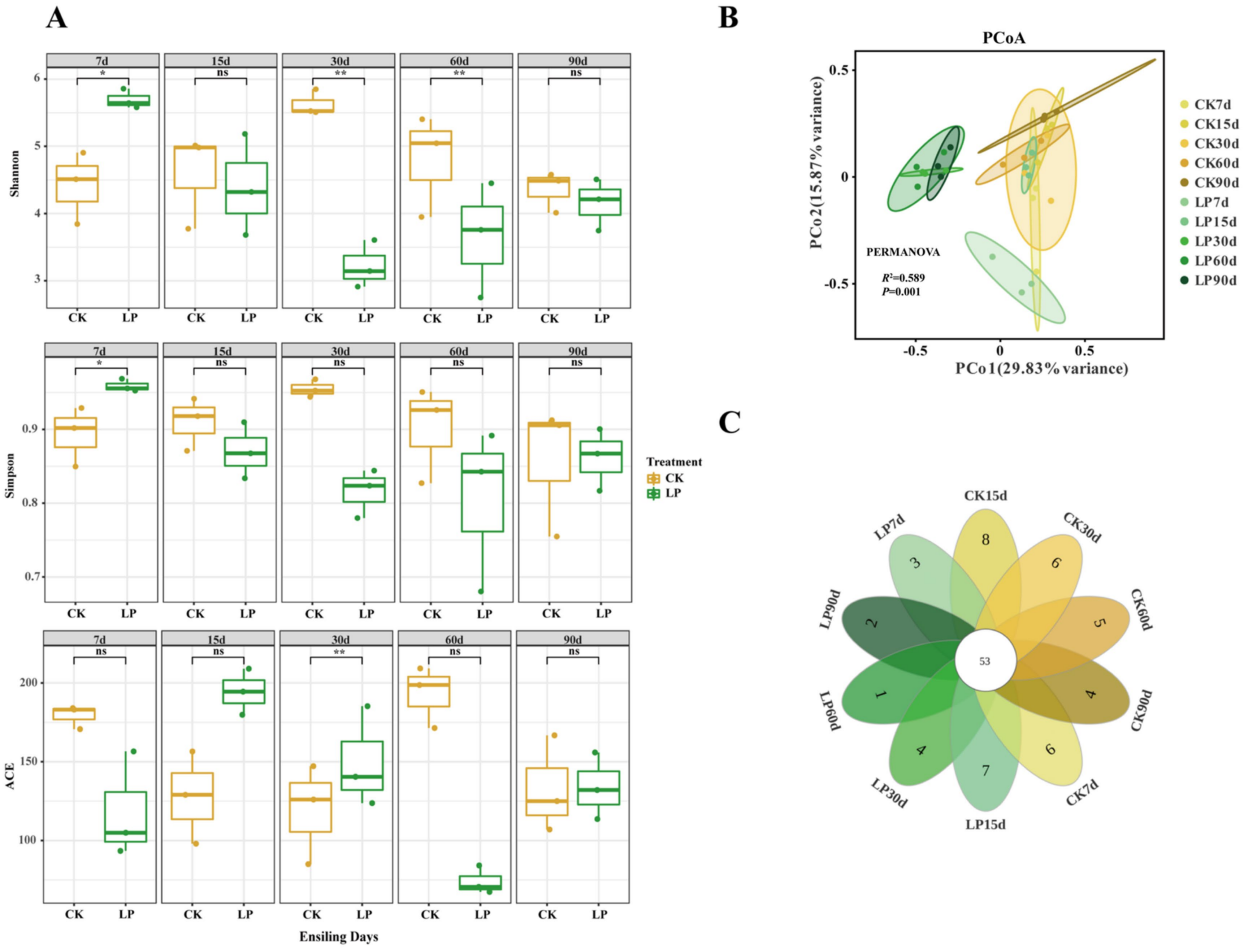
Comprehensive analysis of bacterial communities in WPS silage during ensiling. **(A)** The variations in bacterial community alpha-diversities (Shannon index, Simpson and ACE); **(B)** Principal coordinate analysis (PCoA) of the bacterial communities in WPS silage at the different ensiling days; **(C)** Petals diagram of WPS silage: Individual petals corresponded distinct groups, with the central figure indicating the shared ASVs across all conditions, and the numbers within each petal denoting group-specific ASVs (CK, control with no inoculations; LP, silages inoculated with *Lactiplantibacillus plantarum*; 7d, 7 days of ensiling; 15d, 15 days of ensiling; 30d, 30 days of ensiling; 60d, 60 days of ensiling; 90d, 90 days of ensiling; *p < 0.05; **p < 0.01; ns, no significant effect).

A total of 2,192,816 high-quality reads were identified after denoising and filtering for chimeras in the V3-V4 region of the 16S rDNA. The dilution curve change (Figure A1) and average coverage exceeded 0.99 across all samples (Table A1), indicating the sufficient sequencing data for detailed bacterial community analysis in this study. Reads were classified into 445 ASVs. As shown in Figure 1A, significant differences in biodiversity were observed during the silage fermentation process. The Shannon Index was notably lower in the LP group compared to CK30d and CK60d (*p* < 0.01), while the Simpson Index was significantly higher in the CK group than in LP7d and LP30d (*p* < 0.05). The ACE index was also higher in the CK group compared to the LP30d (*p* < 0.01). In Figure 1B, the principal coordinate analysis (PCoA) based on Bray-Curtis distances assessed the beta diversity among silage samples, showing greater similarity. PCoA1 and PCoA2 axes accounted for 29.83 and 15.87% of the microbial composition variance in silage feed, respectively, totaling 45.7%. PERMANOVA analysis verified significant differences in beta diversity among the groups (*R*^2^ = 0.589, *p* = 0.001). The petals diagram (Figure 1C) illustrated that the shared number of common ASVs between the CK and LP groups at different ensiling days was 53. The number of unique ASVs in the LP group was smaller than that of the CK group at the same ensiling days, with the lowest number at LP60d.

#### Taxonomic and abundance of bacterial populations

**Figure 2:**
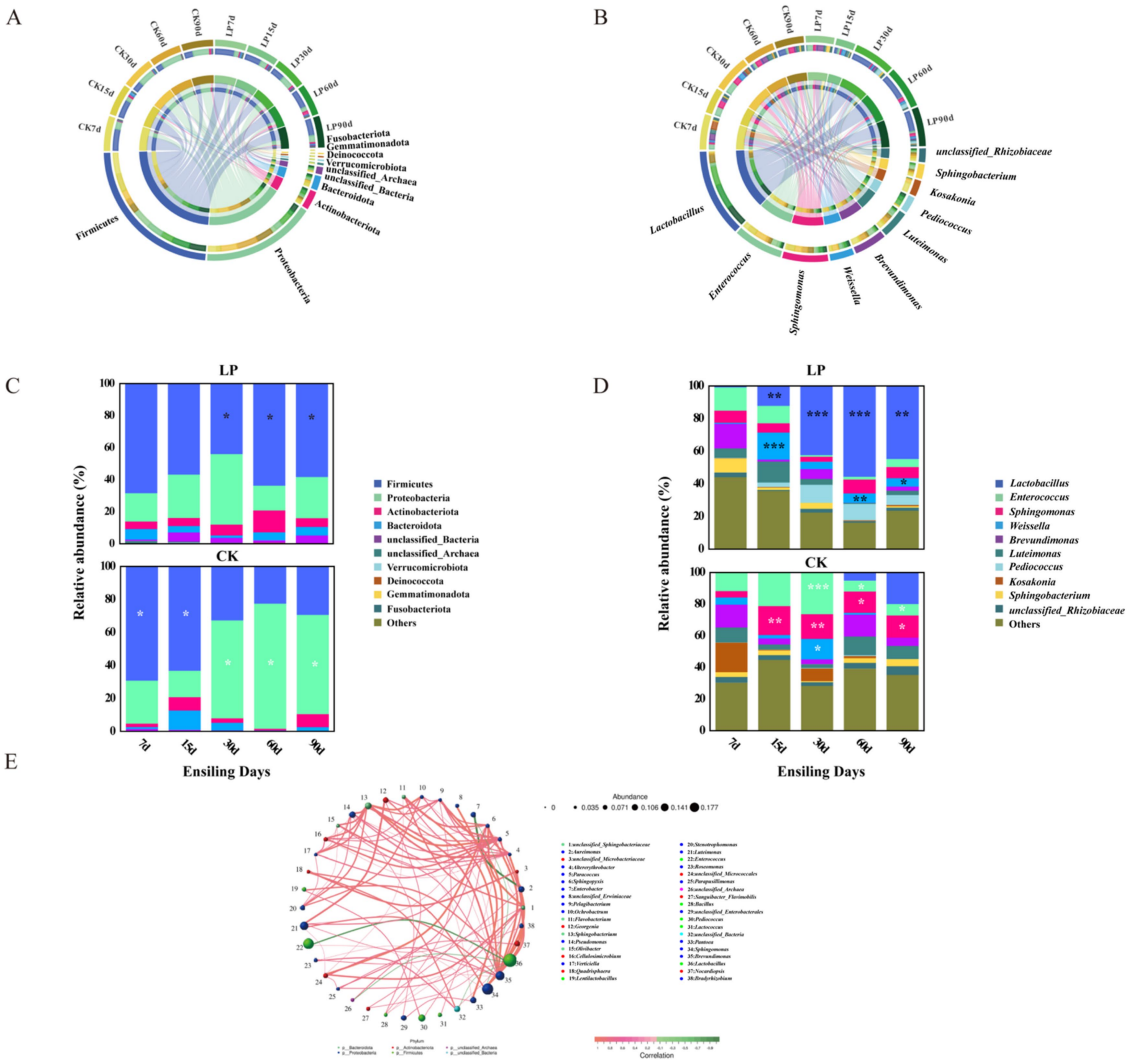
Bacterial community diversities and compositions in WPS silage during ensiling. **(A)** Circos map of bacterial communities at the phylum level during ensiling; **(B)** Circos map of bacterial communities at the genus level during ensiling; **(C)** Stacked bar charts showing relative abundance of bacterial community at phylum level. Differences in relative abundance were analyzed using a two-sided Student’s *t*-test (*p < 0.05; **p < 0.01; ***p < 0.001). White asterisks represented a lower relative abundance of this phylum in LP treatments compared to the CK group, while dark asterisks indicated a higher relative abundance in CK treatments relative to the CK group. **(D)** Stacked bar charts showing relative abundance of bacterial community at genus level. Differences in relative abundance were analyzed using a two-sided Student’s *t*-test (*p < 0.05; **p < 0.01; ***p < 0.001). White asterisks represented a lower relative abundance of this genus in LP treatments compared to the CK group, while dark asterisks indicated a higher relative abundance in CK treatments relative to the CK group. **(E)** Microbial network diagram at the genus level based on Spearman rank correlation analysis. Each circle in the diagram denoted a genus, with its size corresponding to the genus’s relative abundance. Distinct phylums were indicated by various colors. Lines connecting the circles signified statistically significant correlations (*p* < 0.05) between bacterial genus, with red indicating positive and green negative correlations. The line thickness reflected the strength of the correlation coefficient.

As shown in Figure 2A, at the phylum level, Firmicutes and Proteobacteria were the dominant bacterial phyla during the different ensiling times, playing significant roles in the fermentation process of silage (Song et al., 2022). The bacteria within the Firmicutes phylum produce extracellular enzymes that facilitate the breakdown of macromolecules such as proteins, polysaccharides, and lipids into smaller molecules like monosaccharides, amino acids, and fatty acids (Yan et al., 2019). It has also been reported that lactic acid fermentation is primarily driven by bacteria from the Firmicutes phylum (Wang W. et al., 2022). Concurrently, the *Proteobacteria* phylum, which metabolizes organic matter as substrates, is often present in silage feed, similar to the *Actinobacteria* phylum (Mu et al., 2020). The predominant genera identified were *Lactobacillus*, *Enterococcus*, *Sphingomonas*, and *Weissella*, with *Lactobacillus*, *Enterococcus*, and *Weissella* belonging to the order *Lactobacillales*, commonly referred to as LAB. *Sphingomonas* was the exception, not classified within this order (Figure 2B). Firmicutes were more abundant in CK7d (Figure 2C). In the LP groups, their relative abundance increased during the later fermentation stages, likely due to Firmicutes’ ability to thrive in acidic and anaerobic environments (Kang et al., 2021). Except for 15 days of ensiling, the LP groups had a higher relative abundance of Proteobacteria compared to the CK groups at other ensiling days. With the addition of LP, the bacterial community structure changed, and the relative abundance of *Lactobacillus* became the dominant genus in the LP60d group (55.68%) (Figure 2D; Table 2). On the genus level, the bacterial community showed significant correlations, with *Lactobacillus* displaying a negative correlation with *Enterococcus* (*p* < 0.05) and a positive correlation with both *Pediococcus* and *Lentilactobacillus* (*p* < 0.05), while *Enterococcus* displayed a positive relationship with *Pediococcus* and *Lentilactobacillus* (*p* < 0.05) (Figure 2E).

#### Biomarkers analysis

**Figure 3:**
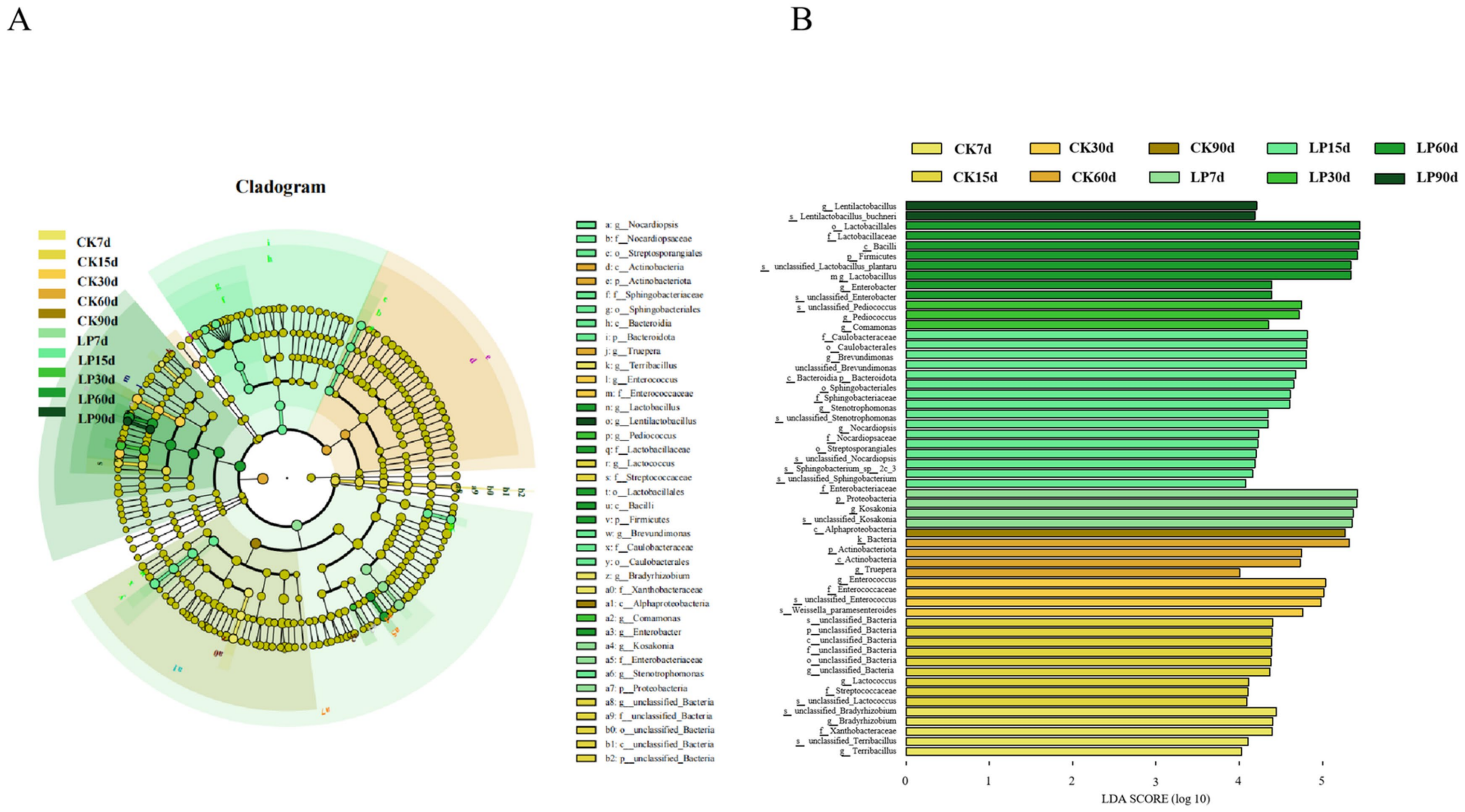
Linear Discriminant Analysis Effect Size (LEfSe) of microbial differences in WPS silage during ensiling. **(A)** Cladogram differences at various phylogenic levels; **(B)** LEfSe analysis with linear discriminant analysis (LDA) score. Discriminative features were identified using LDA score threshold of 4.0, with Kruskal-Wallis and Wilcoxon test significance at *p* < 0.05.

The Linear Discriminant Analysis Effect Size (LEfSe) analysis identified 55 bacterial phyla-to-species as biomarkers in both the CK and LP groups at different ensiling days (Figure 3). At the phylum level, *Proteobacteria* were enriched in the LP7d and Firmicutes were enriched in the LP90d. Significant markers identified in the LP60d group included Lactobacillales (order), Lactobacillaceae (family), *Lactobacillus* (genus), and *unclassified_Lactobacillus_plantarum* (species), all of which are beneficial for silage fermentation. *Lentilactobacillus_buchneri* (species) emerged as the most influential microbial species in the LP90d samples, a bacterium known to enhance the heterofermentation of AA and improve aerobic stability in silage, consistent with the findings reported by Xiao et al. (2023). This was also correlated with a significantly higher AA content in the LP90d compared to other time points.

### Metabolites

#### Metabolites composition, time series analysis, and differentially accumulated metabolites

**Figure 4:**
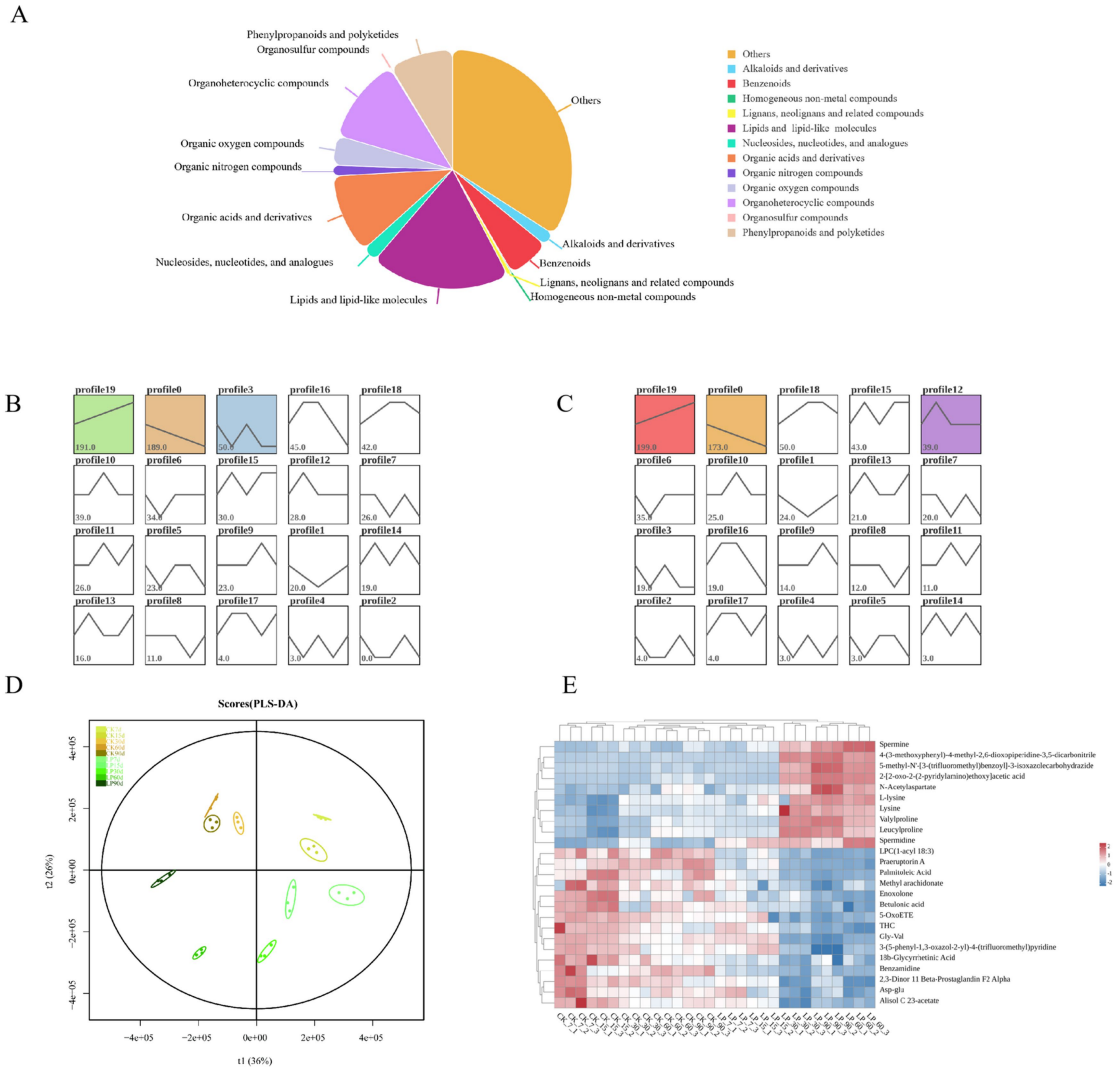
Metabolites in WPS silage during ensiling. **(A)** Pie chart distribution of all metabolites in WPS; **(B)** Time series expression analysis of different ensiling days in CK groups by the Short Time-series Expression Miner algorithm; **(C)** Time series expression analysis of different ensiling days in WPS ensiling with *Lactobacillus plantarum* by the Short Time-series Expression Miner algorithm. The lower left corner number represented the number of metabolites with the trend, profiles with color indicated statistically significant differences in trends (*p* < 0.05); **(D)** Score plots of PLS-DA. X and Y axes indicated the 1st and 2nd components; **(E)** Heatmap of the top 25 differential accumulated metabolites of CK group or LP group at the different ensiling days.

As depicted in Figure 4A, a total of 1,321 chemicals were identified in WPS silage, including 250 lipids and lipid-like molecules, 153 organoheterocyclic compounds, 142 organic acids and its derivatives, and 115 phenylpropanoids and polyketides, among others. Lipids, reported to be the most abundant in WPS silage, potentially contribute to the increase of milk fat content in cows fed with WPS silage (Vargas-Bello-Pérez et al., 2008). Additionally, numerous nutritional unsaturated fatty acids were present in WPS silage, including Oleic acid, linoleic acid, Palmitoleic acid, and Eicosapentaenoic acid. A variety of flavonoid metabolites such as Robinin, Neodiosmin, Icariin, Trifolin, Pelargonidin, and Farrerol were detected, with notable increases in the abundance of Pelargonidin and Farrerol with LP addition compared to the CK group (Figure A2). The study identified 17 α-amino acids and their derivatives, including Lysine and Threonine. The addition of LP significantly increased the abundance of Lysine and L-Threonine, enriching feed nutrition (Figure A2). In both CK and LP groups, three profiles of metabolites exhibited consistent significant trends at five different ensiling durations by time series expression analysis, with the most abundant trends in both groups being continuous upregulation, featuring 191 and 199 metabolites, respectively (Figures 4B,C). The PLS-DA model was used to identify differential metabolites between groups, showing all sample points within Hotelling’s T-squared, indicating a sufficient generalization ability to discriminate between CK and LP samples across various ensiling days (Figure 4D). From 30 days of ensiling, the abundance of Lysine, L-lysine, Spermine, and Spermidine in the LP groups exceeded that in the CK groups, as demonstrated by a correlation heatmap of the top 25 differential metabolites (Table A2; Figure 4E).

#### Differentially metabolites KEGG pathway enrichment

**Figure 5:**
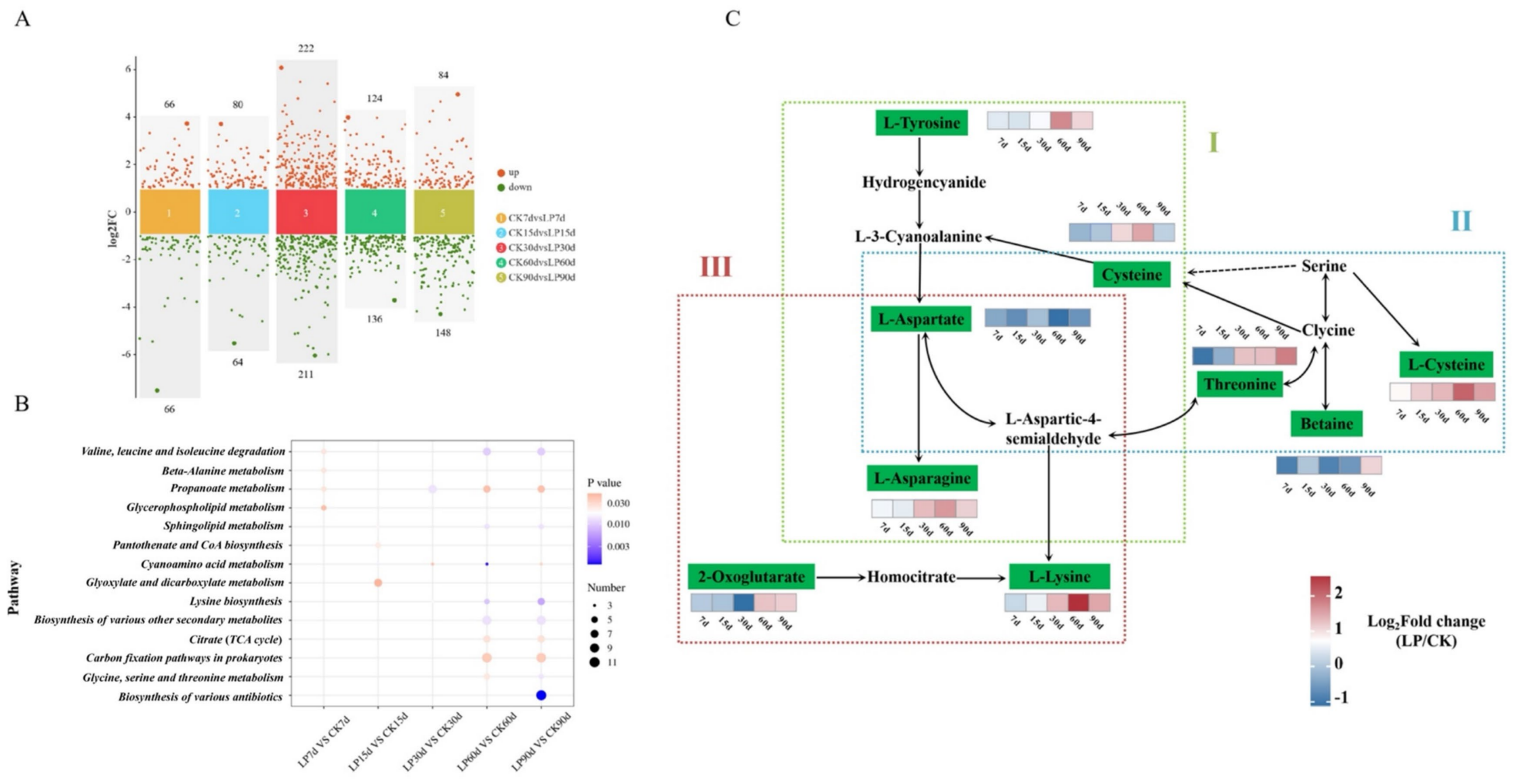
**(A)** Volcano plot analysis of differential metabolites in the CK and LP at the different ensiling days. Points on the volcano plot represented differentially accumulated metabolites (DAMs) with a log2 fold change (log2FC) greater than 1 between the two groups, indicating at least a twofold increase or decrease (*p* < 0.05); **(B)** Dot plot analysis of the KEGG pathway enrichment for significantly different metabolites accumulation in CK vs. LP at the different ensiling days (*p* < 0.05). The x-axis and y-axis, respectively, represented the different groups and the enrichment pathway; **(C)** KEGG metabolic network analysis of the pathways in the silage. Parts I, II, III represented the Cyanoamino acid metabolism, Glycine, Serine, and Threonine metabolism, and Lysine biosynthesis, respectively. The differentially accumulated metabolites (DAMs) were shown in green. The color block represented the fold changes (LP/CK) after log2 conversion, with upregulation in red and downregulation in blue.

The quantity of differentially accumulated metabolites (DAMs) varied between the CK and LP groups as ensiling duration increased, initially rising to a peak at 30d, followed by stabilization from 60 to 90 days (Figure 5A). KEGG pathway enrichment analysis indicated five amino acid metabolism pathways were significantly enriched, “Cyanoamino acid metabolism,” “Glycine, Serine, and Threonine metabolism,” and “Lysine biosynthesis,” with the higher number of enriched pathways at 60d and 90d (Figure 5B). Except for L-Aspartate pathways, the relative concentrations of other amino acids significantly increased with the addition of LP, L-Tyrosine, L-Asparagine, L-Lysine, and L-Cysteine reached their maximum expression at 60d. This suggested that addition of LP in WPS silage significantly enhanced the metabolism of amino acids beneficial to animals (Figure 5C).

#### The relationships among bacterial community, metabolites, and key fermentation quality indicators

**Figure 6:**
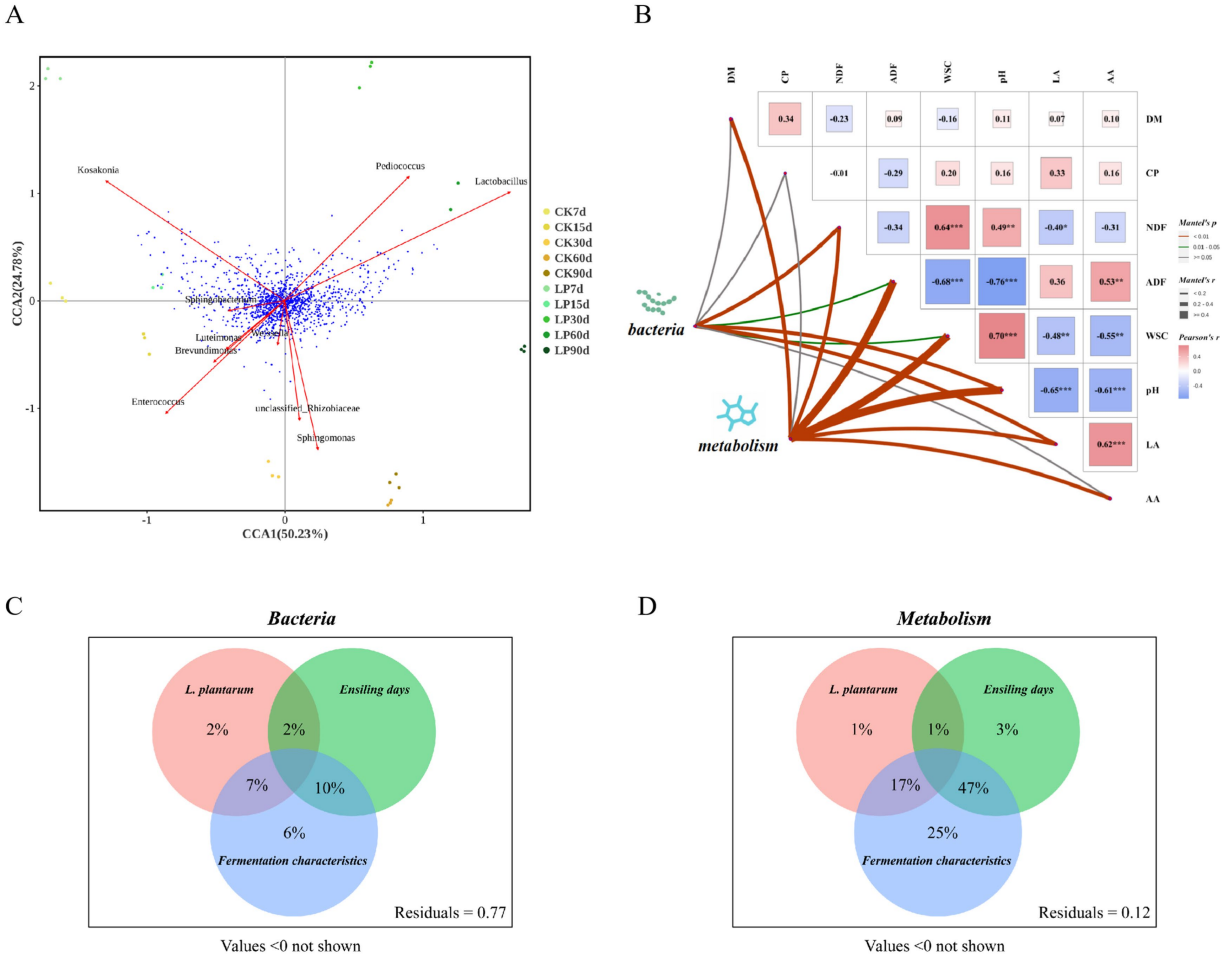
Correlation analysis of fermentative indicators, bacteria and metabolites. **(A)** Canonical correlation analysis (CCA) of bacterial and metabolites in WPS silage. The red vector arrows represented the top 10 bacteria with relative abundance at the genus level, and the blue points represented metabolites; **(B)** The Mantel test correlation plot between bacteria, metabolites and key fermentation quality indicators based on Pearson’s correlation coefficients, with the red and blue squares indicating positive and negative correlation, respectively. The left two points symbolized the matrices for bacteria and metabolites. A thicker line indicated a stronger correlation by a higher Mantel’s r value. The green Line indicated significance with a Mantel’s *p* value between >0.01 and <0.05, while red denoted high statistical significance (*p < 0.05, **p < 0.01, ***p < 0.001) with a Mantel’s *p* value of <0.01; **(C,D)** Variation partitioning analysis (VPA) to determine the effects of addition of *Lactiplantibacillus plantarum*, ensiling days, fermentation characteristics, and interactions between these parameters on the structure of the bacterial communities and metabolites composition. Circles without overlap showed the percentage of variation explained by each factor alone. The overlap region of two or three circles displayed the explanation of variation between two or three of these factors.

Chemical changes in silage feed are intricately linked to bacterial communities, as demonstrated by CCA analysis in WPS silage (Figure 6A). *Lactobacillus* was found to be positively correlated with *Pediococcus* and negatively with *Enterococcus*, consistent with findings in Figure 2E. Additionally, *Luteimonas* exhibited a strong positive correlation with *Brevundimonas*. Points from the LP60d group clustered nearest to *Lactobacillus*, indicating a strong association at 60 days of ensiling with LP. The proximity of a multitude of metabolites to *Lactobacillus* suggested a significant connection between the relative abundance of *Lactobacillus* and these metabolites’ levels. Other bacteria such as *Enterococcus* and *Pediococcus* also showed a dense clustering of metabolite points, suggesting their potential impact on the metabolomic spectrum. Furthermore, based on the results of the Mantel test (Figure 6B), no significant correlations were observed between DM and CP concentrations. A significant negative correlation was noted between pH and the concentrations of LA and AA (*p* < 0.01). NDF concentration showed a highly significant positive correlation with WSC concentration (*p* < 0.001) and pH (*p* < 0.01), but a significant negative correlation with LA concentration (*p* < 0.05). ADF concentration was significantly positively correlated with AA concentration (*p* < 0.01) and negatively with pH and WSC concentration (*p* < 0.01). The bacterial community showed positive correlations with pH, LA concentration, and NDF (*p* < 0.01), and with ADF concentration and WSC (*p* < 0.05). The metabolome did not correlate with CP concentration but had strong correlations with other indicators, particularly pH (*p* < 0.01). A VPA analysis (Figures 6C,D) was performed to determine the effects of the addition of *Lactiplantibacillus plantarum*, ensiling days, and fermentation characteristics on the structures of the bacterial communities and metabolite composition. This analysis indicated that the largest share of the variation in bacterial community structure (47%) and metabolite composition (25%) could be attributed to the interaction between fermentation characteristics and ensiling days. The addition of *Lactiplantibacillus plantarum* also had significant effects, explaining 17% of the variation in metabolite composition and 7% in bacterial community structure. These findings demonstrate the complex interactions that drive the microbiological and chemical dynamics of silage fermentation.

## Discussion

The nutritional quality of forage is influenced by several factors including plant genotype, sowing density, harvesting season, and water and fertilizer conditions. In this study, the relatively high levels of CP and EE in the WPS may be attributed to the use of a soybean variety specifically bred for feed purposes, along with optimal sowing densities and fertilization during the cultivation phase. The WSC content is a critical factor influencing silage fermentation, playing a pivotal role in ensuring quality fermentation when its content exceeds 5%. In this study, the WSC content of WPS before ensiling was found to be 3.84%, indicating that ensiling soybeans alone is difficult and challenging without the addition of additives. The consumption of sugar by microorganisms during the fermentation process leads to the production of lactic acid, which reduces the contents of DM and WSC. With the addition of LP, there was a significant increase in the DM, CP, and WSC content of the silage (*p* < 0.01) and a significant decrease in pH (*p* < 0.01). The pH of silage is a traditional and critical indicator for evaluating fermented feeds. McDonald et al. (1991) identified a pH of 4.20 as critical for high-quality silage conservation, while Ahmadi et al. (2019) noted that a pH range of 3.8 to 4.2 is optimal. In the LP group, the pH was reduced to below 4.2 after 60 days of ensilage, indicating high-quality silage. Previous studies have shown that low levels of ADF and NDF are associated with higher quality feeds and higher dry matter intake (Ni et al., 2014). Notably, the CK and LP groups showed lower contents of ADF and NDF at 60 days, indicating they have a higher feed value compared to other fermentation periods. The decrease in crude fiber content could be attributed to the enzymatic activity of microorganisms, which break down the fiber during the fermentation process (Chen et al., 2022). The EE content in the CK group showed a significant increasing trend with the increase of ensiling days (*p* < 0.01), with the highest content (3.49%) at 60 days of silage fermentation being more than that in the CK group. This may be due to the microbial community change and reduction of lipolytic bacteria with the addition of LP. The addition of LP caused a significant increase in propionic acid (PA) levels. PA is known to effectively inhibit the growth of molds and yeasts, thereby reducing the occurrence of secondary fermentation (Gheller et al., 2021). PA can also promote the growth of LAB, modulating the ensiling process to minimize protein degradation. The undesirable BA was detected in the CK60d and CK90d, while it was absent in the LP groups. As the ensiling time increased, the aerobic stability of the silage progressively improved (Kung et al., 2018). The addition of LP significantly enhanced the aerobic stability and fermentation quality of WPS silage when compared to the CK group. Similar effect of LP on fermentation quality and microbial dynamics have been extensively reported in alfalfa silage systems. For instance, studies have shown that LP addition enhances lactic acid production, improves aerobic stability, reduces BA levels, and lowers silage pH by promoting rapid acidification, thereby suppresses undesirable microorganisms in alfalfa-based silage formulations (Yang et al., 2020; Li et al., 2021; Peng et al., 2024). These findings provide contextual support for the efficacy of LP observed in WPS silage in the present study. Such cross-species consistency highlights LP’s broad-spectrum utility as a microbial inoculant in legume-based silage and reinforces the potential of WPS as a valuable forage resource when appropriately inoculated.

The results highlight the substantial impact of *Lactiplantibacillus plantarum* on microbial structures during fermentation, evident from the distinct microbial community clustering between the CK and LP groups at different ensiling days. The significant reduction in bacterial diversity and species abundance with LP treatment, as indicated by the Shannon, Simpson, and ACE indexes, suggests an effective modulation of the bacterial community by LP. This could be linked to the antibacterial action of LP, which may inhibit the growth of harmful microorganisms, thereby improving the overall fermentation process (Dinev et al., 2018; He et al., 2021). The marked difference in the unique ASVs numbers between the CK and LP groups, especially at LP60d, underscores the potential of LP in selectively suppressing undesirable bacteria while possibly promoting beneficial ones. This selective antibacterial action could play a critical role in enhancing the stability and quality of silage, making LP an important factor in silage management and microbial ecology. The fermentation level of silage is significantly determined by the epiphytic microorganisms on the forage material used for ensiling (Lu et al., 2023). In addition to the direct impact of *Lactiplantibacillus plantarum*, it is important to consider the potential interactions between LP and native epiphytic microbes such as *Weissella*, which may also contribute to early lactic acid production and pH decline. *Weissella* genus are known to be active in the early stage of fermentation due to their ability to grow in moderately acidic environments, possibly supporting or even synergizing with LP’s acidification process (Kong and Park, 2019). Thus, the observed reduction in pH might result from combined microbial activities rather than solely LP’s dominance. Future studies incorporating microbial interaction models or co-culture inoculation trials may further clarify these relationships.

The significant increase in *Lactobacillus* abundance in the LP group, as demonstrated by the t-test, highlights its crucial role in the silage fermentation process. *Enterococcus*, *Weissella*, and *Pediococcus* genera, known for their early production of LA in silage feed, displayed high relative abundance in both CK and LP groups, contributing beneficially to silage fermentation. The initial higher pH levels likely favored the growth of *Enterococcus*, *Pediococcus*, and *Weissella*, which have relatively low tolerance to lower pH environments created by LA production. This environment becomes more favorable for *Lactobacillus* growth as the fermentation progresses. The relative abundance of *Enterococcus* in the LP group declined from 15 to 30 days of ensiling, earlier than in the CK group, possibly due to the rapid decrease in pH facilitated by increased lactic acid content with LP addition. Similar suppression of *Enterococcus* has also been observed in alfalfa silage treated with *Lactiplantibacillus plantarum* (Li et al., 2018; Zhang et al., 2023). This suppression is primarily achieved by enhancing the acidification environment of the silage (Na et al., 2022). This consistency across forage types further supports LP ‘s role as a broadly effective silage inoculant for modulating microbial communities. This hastens the acidification process, improving the microbial community and enhancing the quality of the silage. Furthermore, the presence of *Sphingomonas* and *Luteimonas*, related to the enhancement of cellulose decomposition, correlated with the decrease in ADF and NDF contents as ensiling time increased (Dumova and Kruglov, 2009). The detection of *Luteimonas* in WPS silage appears novel, as no prior studies have reported this genus in soybean silage fermentation. Additionally, *unclassified_Rhizobiaceae* were also observed, possibly originating from epiphytic bacteria on soybean plants, as *Rhizobiaceae* have been noted in the silage of other legume forages (Huffman et al., 2023). The microbial system of silage fermentation represents a complex network (Sun et al., 2021), essentially a competitive process between LAB and undesirable microbes. High-quality silage is characterized by a simplified bacterial structure and the proliferation of beneficial microbes (Bai et al., 2021). The WPS silage in the LP60d exhibited the characteristics of high-quality silage feed, demonstrating improved fermentation quality. This suggests that targeted microbial interventions, such as the addition of specific LAB strains, can effectively manipulate the microbial ecosystem to favor beneficial processes, thereby enhancing the overall quality and stability of the silage. This approach not only improves the nutritional value but also potentially extends the storage life of silage, making it a more viable feed option for livestock.

Flavonoids, as principal phenolic substances of plant secondary metabolites, play a beneficial role in health due to their antioxidant, anti-inflammatory, antimicrobial, antimutagenic, and anticancer properties (Proestos et al., 2006). Xu et al. (2020) identified several flavonoids in red clover silage as differential metabolites between control and inoculant treatments, suggesting a regulatory effect of the inoculant LP on flavonoid content during ensiling. This indicates that LP addition may enhance the anti-inflammatory and antioxidant capabilities of silage, potentially benefiting animal health. Lysine is an essential amino acid for animal growth and maintenance, enhancing protein absorption and utilization (Khwatenge et al., 2020), while Threonine plays an important role in immunity in animal feed production (Liu et al., 2022). This enhanced understanding of how LP affects the metabolic profile of silage can help improve the nutritional quality and health benefits of silage. The results from the VPA revealed that when considered individually, fermentation characteristics explained the highest percentage of variation observed in the bacterial communities and metabolite composition, accounting for 6 and 25%, respectively. However, interactions between fermentation characteristics and ensiling days (10 and 47%), as well as between fermentation characteristics and the addition of *Lactiplantibacillus plantarum* (7 and 17%), were identified. Notably, the interaction between fermentation characteristics and ensiling days emerged as a more significant driver of bacterial communities and metabolites composition in silage. This analysis fills the informational gap between microbial communities and metabolites in leguminous forages, particularly soybean, and is essential for selecting beneficial inoculants. The interplay of bacterial dynamics and metabolic profiles underscores the complexity of the silage fermentation process and highlights the potential for targeted microbial interventions to enhance silage quality and nutritional value.

This study was conducted under controlled laboratory conditions, which may not entirely represent the complexity of field-scale silage fermentation. As such, further investigations under practical on-farm settings are warranted to validate the applicability of our findings.

## Conclusion

In summary, the addition of LP to WPS significantly increased the DM, CP, WSC, and LA content of silage, and significantly decreased pH, significant increase of LA concentration of WPS silage enhanced the relative abundance of beneficial bacteria like *Lactobacillus*, inhibited undesirable microbes, and improved bacterial community and fermentation quality. Two hundred and fifty types of lipids and lipid molecules with beneficial effects on milk fat content increase, various nutritional unsaturated fatty acids such as oleic acid and linoleic acid, as well as beneficial secondary metabolites such as flavonoids were detected in WPS with addition of LP through metabolomics analysis. Notably, Lysine metabolism was influenced by the addition of *Lactiplantibacillus plantarum*, which significantly boosted the abundance of this essential amino acid, improving the nutritional value of the silage. At the late stage of fermentation for 60 days, the fermentation quality, beneficial microorganisms, and advantageous metabolites of silage have significant advantages in WPS silage with addition of LP, the findings would provide technical support for a high-quality silage soybean forage resource.

## Data Availability

The raw reads were deposited in the National Center for Biotechnology Information (NCBI) database under accession number PRJNA1085090.

## Footnotes

^1^ https://www.arb-silva.de/

^2^ http://www.kegg.jp/kegg/pathway.html

^3^ https://www.omicshare.com/tools
