# Supplementary figures and images for: Impact of Lactiplantibacillus plantarum on the fermentation quality, nutritional enhancement, and microbial dynamics of whole plant soybean silage
Source: Front Microbiol. 2025 May 19;16:1565951. doi: 10.3389/fmicb.2025.1565951 (PMC12127759; doi:10.3389/fmicb.2025.1565951)

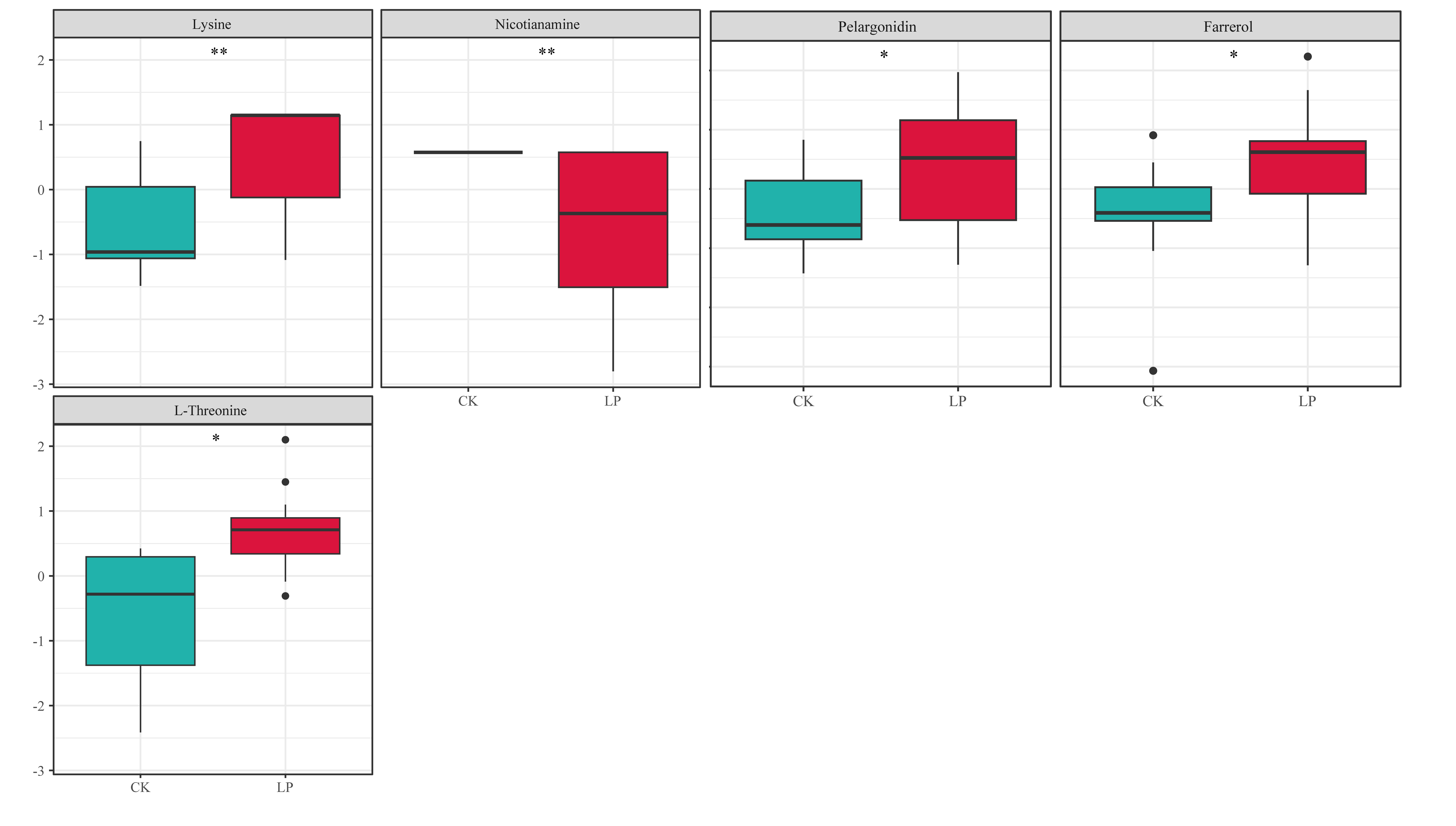

Supplement: Supplementary file 2 [file Image_2.tif]
